# 2D Nitrogen‐Doped Graphene Materials for Noble Gas Separation

**DOI:** 10.1002/smll.202408525

**Published:** 2024-11-06

**Authors:** Veronika Šedajová, Min‐Bum Kim, Rostislav Langer, Gobbilla Sai Kumar, Lili Liu, Zdeněk Baďura, James V. Haag, Giorgio Zoppellaro, Radek Zbořil, Praveen K Thallapally, Kolleboyina Jayaramulu, Michal Otyepka

**Affiliations:** ^1^ Regional Centre of Advanced Technologies and Materials Czech Advanced Technology and Research Institute (CATRIN) Palacký University Olomouc Šlechtitelů 27 Olomouc 783 71 Czech Republic; ^2^ Energy and Environmental Directorate, Pacific Northwest National Laboratory Richland WA 99352 USA; ^3^ IT4Innovations VŠB–Technical University of Ostrava 17. listopadu 2172/15 Ostrava‐Poruba 708 00 Czech Republic; ^4^ Hybrid Porous Materials Laboratory, Department of Chemistry Indian Institute of Technology Jammu Jammu and Kashmir 181221 India; ^5^ Nanotechnology Centre CEET VŠB‐Technical University of Ostrava 17. listopadu 2172/15 Ostrava‐Poruba 708 00 Czech Republic

**Keywords:** 2D materials, defect engineering, noble gas separation, selectivity, symmetry‐adapted perturbation theory (SAPT), xenon

## Abstract

Noble gases, notably xenon, play a pivotal role in diverse high‐tech applications. However, manufacturing xenon is an inherently challenging task, due to its unique properties and trace abundance in the Earth's atmosphere. Consequently, there is a pressing need for the development of efficient methods for the separation of noble gases. Using mild fluorographene chemistry, nitrogen‐doped graphene (GNs) materials are synthesized with abundant aromatic regions and extensive nitrogen doping within the vacancies and holes of the aromatic lattice. Due to the organized interlayer “nanochannels”, nitrogen functional groups, and defects within the two‐dimensional (2D) structures, GNs exhibits effective selectivity for Xe over Kr at low pressure. This enhanced selectivity is attributed to the stronger binding affinity of Xe to GN compared to Kr. The adsorption is governed by London dispersion forces, as revealed by theoretical calculations using symmetry‐adapted perturbation theory (SAPT). Investigation of other GNs differing in nitrogen content, surface area, and pore sizes underscores the significance of nitrogen functional groups, defects, and interlayer nanochannels over the surface area in achieving superior selectivity. This work offers a new perspective on the design and fabrication of functionalized graphene derivatives, exhibiting superior noble gas storage and separation activity exploitable in gas production technologies.

## Graphical abstract

Synthesized nitrogen‐doped graphene (GNs) materials via reaction of graphite fluoride with NaN_3_ feature interlayer nanochannels, nitrogen functional groups, and defects in their two‐dimensional (2D) structure. The highly nitrogen doped graphene GN‐1 demonstrates proficient Kr storage and superior selectivity for Xe/Kr parting at low pressure, highlighting its potential for gas separation applications.

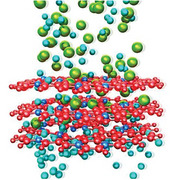

## 1 Introduction

Argon (Ar), krypton (Kr), and namely xenon (Xe) are in demand for various industrial applications such as medical imaging and diagnostics, anesthesia, aerospace propulsion, light bulbs, semiconductors manufacturing, and chemistry.^[^ ^1^ ^]^ Commercially, pure xenon (a rare atmospheric element occurring at 0.08 ppm) is obtained by cryogenic fractional distillation of air and consecutive purification.^[^ ^2^ ^]^ Such a process is economically extremely demanding and the energy cost associated with the production of 1 kg of Xe is, therefore, three orders of magnitude higher than the cost for the production of steel.^[^ ^3^, ^4^ ^]^ The production of xenon necessitates the development of efficient and sustainable strategies for efficient noble gas separations. Recently the potential of porous materials has been identified for the separation and purification of gas mixtures through physisorption.^[^ ^5^, ^6^, ^7^, ^8^, ^9^, ^10^, ^11^ ^]^ Traditional porous materials like zeolites, clays, and activated carbon can be used for gas separation. Zeolites and activated carbon have also been tested for parting Xe and Kr in air separation units or Xe purification.^[^ ^12^, ^13^ ^]^ However, these materials display low capacity and selectivity. Our research team is diligently exploring metal‐organic frameworks (MOFs) and their hybrids for the adsorption and separation of noble gases, especially xenon (Xe).^[^ ^14^, ^15^, ^16^, ^17^, ^18^, ^19^ ^]^ We investigate these materials under static and dynamic conditions, capitalizing on their expansive surface area, customizable functional pores, unoccupied metal sites, and hydrophobic components, all of which contribute to substantial isosteric heat of interaction.^[^ ^16^, ^17^, ^18^, ^20^, ^21^ ^]^ However, similar to other adsorption‐based separation methods, achieving an optimal equilibrium between gas uptake and selectivity is crucial for practical, real‐world applications. Furthermore, the stability of MOF is a challenging task for industry‐relevant applications in noble gas storage and separation.^[^ ^1^, ^22^ ^]^

In gas separation, besides porous materials, scientific advancements have led to the exploration of various other materials. Notably, two‐dimensional (2D) materials have emerged as key players in gas separation technology.^[^ ^23^, ^24^, ^25^ ^]^ Their significance lies in their high surface‐to‐volume ratio and compatibility with device design, making them ideal for nanodevice fabrication.^[^ ^26^ ^]^ Furthermore, these 2D materials facilitate the creation of high‐performance separation due to their planar laminar flow structure, and adjustable surface features.^[^ ^20^ ^]^ As a result, they stand as vital components for advanced molecular separation techniques. The exceptional permeability of 2D materials in the gas separation technology opens pathways for ultra‐fast and highly selective separation of water and gases. In essence, the evolution of materials, including 2D nanostructures, continues to revolutionize the field of gas separation, promising efficient and precise molecular separation processes.^[^ ^27^ ^]^

Among 2D materials, graphene and its derivatives including, e.g., graphene oxide (GO), have captured the attention of numerous researchers in recent years.^[^ ^28^ ^]^ GO and reduced GO have already been utilized in various gas storage and separation applications.^[^ ^29^, ^30^, ^31^ ^]^ However, their performance has been subpar due to the presence of functional groups primarily on the edges rather than the planar surface.^[^ ^32^, ^33^, ^34^ ^]^ This results in randomly stacked adjacent nanosheets, forming disordered interlayer “nanochannels” for mass transport.^[^ ^35^, ^36^, ^37^ ^]^ While this interlayer stacking allows the passage of larger molecules, enabling the selective separation of large molecular compounds, it also permits small molecules to move through the channels.^[^ ^38^ ^]^ Predominantly, this structural characteristic significantly affects their efficiency in gas separation processes.^[^ ^39^ ^]^ However, the strategic introduction of reactive chemical moieties uniformly spread across the graphene basal surface is essential to facilitate an efficient and systematic approach to address challenges related to the controlled placement of functional groups on the plane and the creation of significant defects, both crucial for effective gas storage and separation. One effective method involves substitutional doping, where nitrogen atoms are incorporated into the graphene lattice.^[^ ^40^ ^]^ Substitutional doping modifies the hexagonal lattice by replacing carbon atoms with dopants such as N, B, P, or S. This disrupts the sp^2^ hybridization, causing significant changes in graphene's electronic properties (such as plasmon mobility^[^ ^41^, ^42^ ^]^ or charge transfer^[^ ^43^, ^44^ ^]^). Therefore, the doping will influence any further interactions, including with noble gases.^[^ ^45^ ^]^ It is important to note that different gases and adsorption surfaces can result in substantial variations in adsorption potentials and binding energies.

Here, we synthesized a nitrogen‐doped graphene (GN) materials using fluorographene chemistry.^[^ ^46^, ^47^, ^48^, ^49^ ^]^ The synthetic procedure under solvothermal conditions in DMF solvent yields nitrogen doping and unique structural features of GN (material denoted as GN‐1) including remarkable interlayer spacing and formation of tetrahedral (sp^3^) C─C bonds. Due to the organized interlayer “nanochannels”, nitrogen functional groups, and defects within its 2D structure, GN‐1 exhibits superior selectivity for Xe over Kr separation at low pressure. Theoretical calculations show that the enhanced selectivity is caused by a stronger binding energy of Xe with respect to Kr atoms to GN‐1. The symmetry‐adapted perturbation theory (SAPT) analysis highlights the dominant contribution of London dispersion forces to the interaction of noble gas to GN‐1. Moreover, the energy difference between Kr and Xe binding to GN increases with a higher percentage of N atoms in the graphene lattice. Therefore, we also prepared other nitrogen‐doped graphene derivatives (GN‐2, GN‐3, GN‐4, and GN‐5) using varying amounts of sodium azide in the eco‐friendly solvent Tamisolve which differ in nitrogen content, surface area, and pore sizes. Analysis of these materials confirms that the remarkable selectivity to Xe/Kr is caused by nitrogen functional groups, defects, and interlayer nanochannels rather than the surface area.

## 2 Results and Discussion

**Scheme 1:**
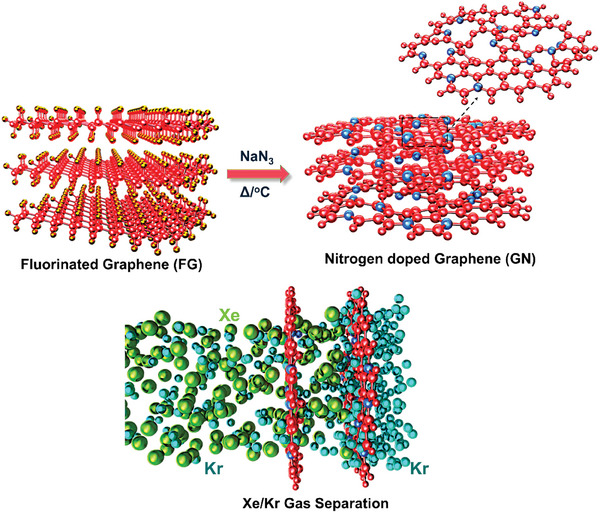
Schematic illustration of the synthesis of nitrogen‐doped graphene (GN) from fluorinated graphene under solvothermal conditions. The resulting GN exhibits effective xenon (Xe) storage and remarkable selectivity for Xe over Kr.

**Figure 1:**
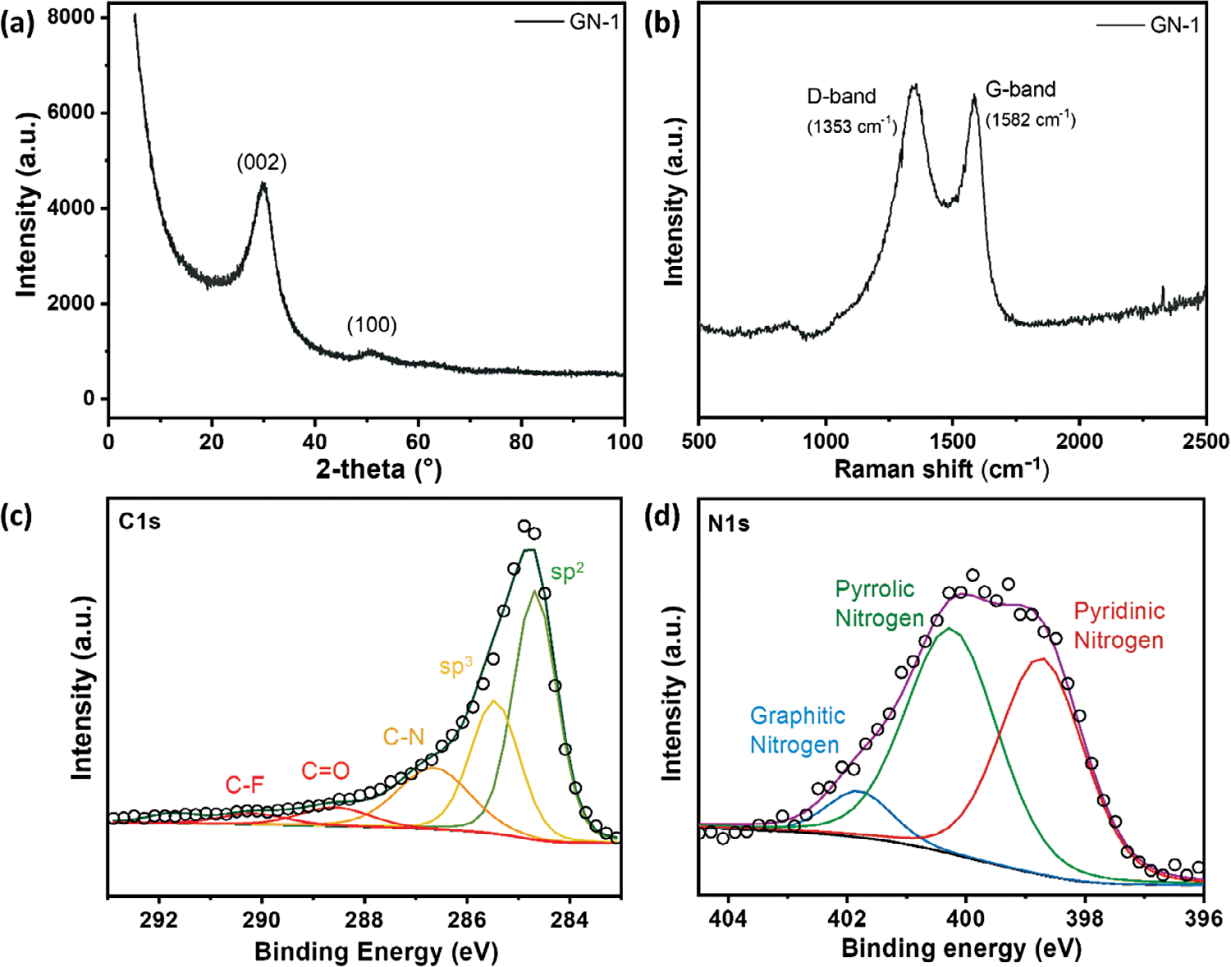
GN‐1 powder XRD pattern a), Raman spectrum b), and high‐resolution XPS spectra of C1s c) and N1s d) with deconvolution.

**Figure 2:**
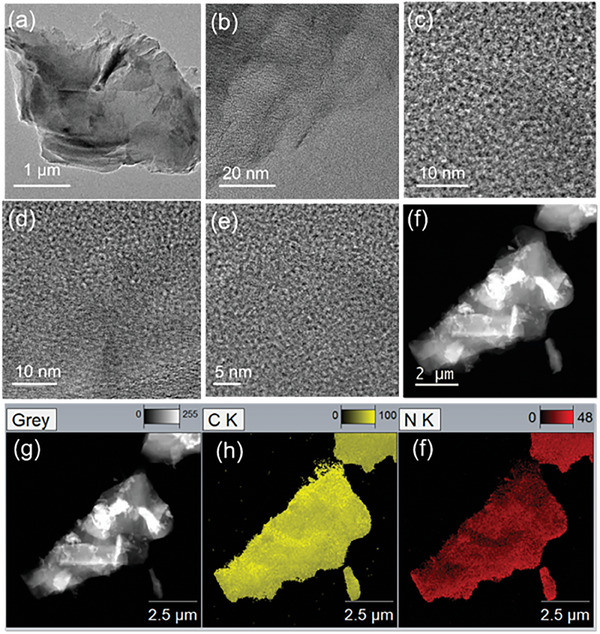
Microscopic analysis of GN‐1 a–e). HRTEM image of GN‐1 shows its layered nature with different magnifications. Elemental mapping demonstrates the homogenous distribution of all corresponding elements, f,g) HAADF image, h) carbon, i) nitrogen.

Herein, we synthesized GNs using up‐scalable and controllable fluorographene chemistry. GN‐1 was prepared from exfoliated fluorographite (FG) by reaction with NaN_3_ under solvothermal conditions in dimethylformamide (DMF)as illustrated in **Scheme** **1**.^[^ ^46^ ^]^ As previously reported, the reaction is initiated by the N_3_^−^ anion via a nucleophilic attack on carbon radical defects. This leads to the elimination of fluorine and the release of nitrogen gas N_2_.^[^ ^46^ ^]^ The phase purity of dried GN‐1 was verified using powder X‐ray diffraction (PXRD). The PXRD pattern exhibited distinct and rather wide peaks at 30.4° and 50.9°, corresponding to (002) and (100) reflections, respectively (**Figure** **1**a). The PXRD indicates on rather disordered graphene structure typical for defective graphene and graphene derivatives. Fourier‐transform infrared spectroscopy (FT‐IR) shows that the FG peaks at 1200 and 1305 cm^−1^ attributed to CF and CF_2_ groups (Figure S1, Supporting Information),^[^ ^46^ ^]^ disappear in GN‐1. On the other hand, a new band at 1580 cm^−1^ can be attributed to aromatic carbon sp^2^, and 1400 cm^−1^ to pyridinic nitrogen substitution in GN‐1. The Raman spectrum of GN‐1 exhibits prominent peaks corresponding to the D‐band (1353 cm^−1^) and G‐band (1582 cm^−1^). The band ratio I_D_/I_G_ = 1.49 indicates the presence of a significant number of sp^3^ carbons and defects (Figure 1b). Moreover, these results are in good agreement with the previously published report, where the in‐depth analysis of the Raman data revealed the presence of C─C tetrahedral bonds between the layers, leading to the holey structure and the presence of channels in the material.^[^ ^46^ ^]^ The X‐ray photoelectron spectroscopy (XPS) survey spectrum of GN‐1 reveals peaks corresponding to carbon, nitrogen, oxygen, and fluorine atoms, with atomic percentages of 77.6%, 17.5%, 2.7%, and 2.2%, respectively (Figure S2, Supporting Information; **Table** **1**). The C 1s signal can be deconvoluted into five peaks representing different carbon bonding states: C─C (sp^2^), C─C (sp^3^), nitrogen‐bonded carbon at 286.8 eV, and oxygen and fluorine bonded carbon at 288.7, and 290.3 eV, respectively (Figure 1c). The detected oxygen signals primarily originate from small amounts of oxygen‐containing functional groups originating from solvent and ambient atmosphere, and small traces can be included in the C─N band area as well, in the form of a C─O bond. In the high‐resolution N 1s spectrum, three peaks were identified at 398.6, 400.8, and 401.9 eV, corresponding to pyridinic, pyrrolic, and graphitic nitrogen atoms, respectively (Figure 1d). The high‐resolution transmission electron microscopy (HRTEM) analysis of GN‐1 at different magnifications reveals a bundle of randomly organized layers, interspersed with irregularly arranged features at varying depths (**Figure** **2**a–e). Besides, in scanning transmission electron microscopy (STEM) images, a consistent presence of carbon and nitrogen elements can be observed throughout the entire sample, indicating the uniform dispersion of these elements, including small impurities of unreacted fluorine and environmental impurities of oxygen (Figure 2f–i). The comprehensive characterization, encompassing Raman bands, XRD, and XPS analysis, establishes that GN‐1 displays a disordered structure featuring randomly developed tetrahedral C─C bonds. The prevalence of pyridinic and pyrrolic nitrogen atoms corresponds to the existing vacancies in the material. This investigation unveils a fibrous micro/meso network‐like structure within GN‐1, marked by a disorderly arrangement of interlayer spacing and the presence of defects in the network.^[^ ^50^ ^]^ To emphasize the influence of nitrogen content in graphene networks, we also synthesized four nitrogen‐doped graphitic carbon materials (GN‐2, GN‐3, GN‐4, and GN‐5) with varying nitrogen source sodium azide as 0.2, 0.5, 1.0, and 2.0 grams respectively. This synthesis was achieved using the same precursor, fluorographite (FG), utilizing the eco‐solvent Tamisolve and by varying the amount of sodium azide under solvothermal conditions (detailed experimental procedures can be found in the Supporting Information). To gain insights into the structural organization and understand existing vacancies and morphological properties, we conducted careful characterization using various techniques including FT‐IR, PXRD, Raman spectroscopy, and Field Emission Scanning Electron Microscopy (FE‐SEM) analysis (as shown in Figures S3–S7, Supporting Information).

**Table 1:** Elemental analysis, textural parameters of resultant nitrogen doped graphene (GN) samples.

Sample Code	Carbon [At. %]	Nitrogen [At. %]	Oxygen [At. %]	Fluorine [At. %]	I_D_/I_G_	BET Surface Area [m^2^ g^−1^]	Total Pore Volume [cm^3^ g^−1^]	Pore Size [Å]
GN‐1	77.6	17.5	2.7	2.2	1.49	32	0.040	17
GN‐2	71.1	6.6	7.5	14.8	1.08	311	0.176	10–20
GN‐3	72.1	10.5	6.7	10.7	1.08	171	0.107	12–25
GN‐4	74.4	12.6	6.6	6.4	1.10	41	0.045	18–26
GN‐5	69.4	19.6	6.5	4.4	1.14	19	0.036	250–350

**Figure 3:**
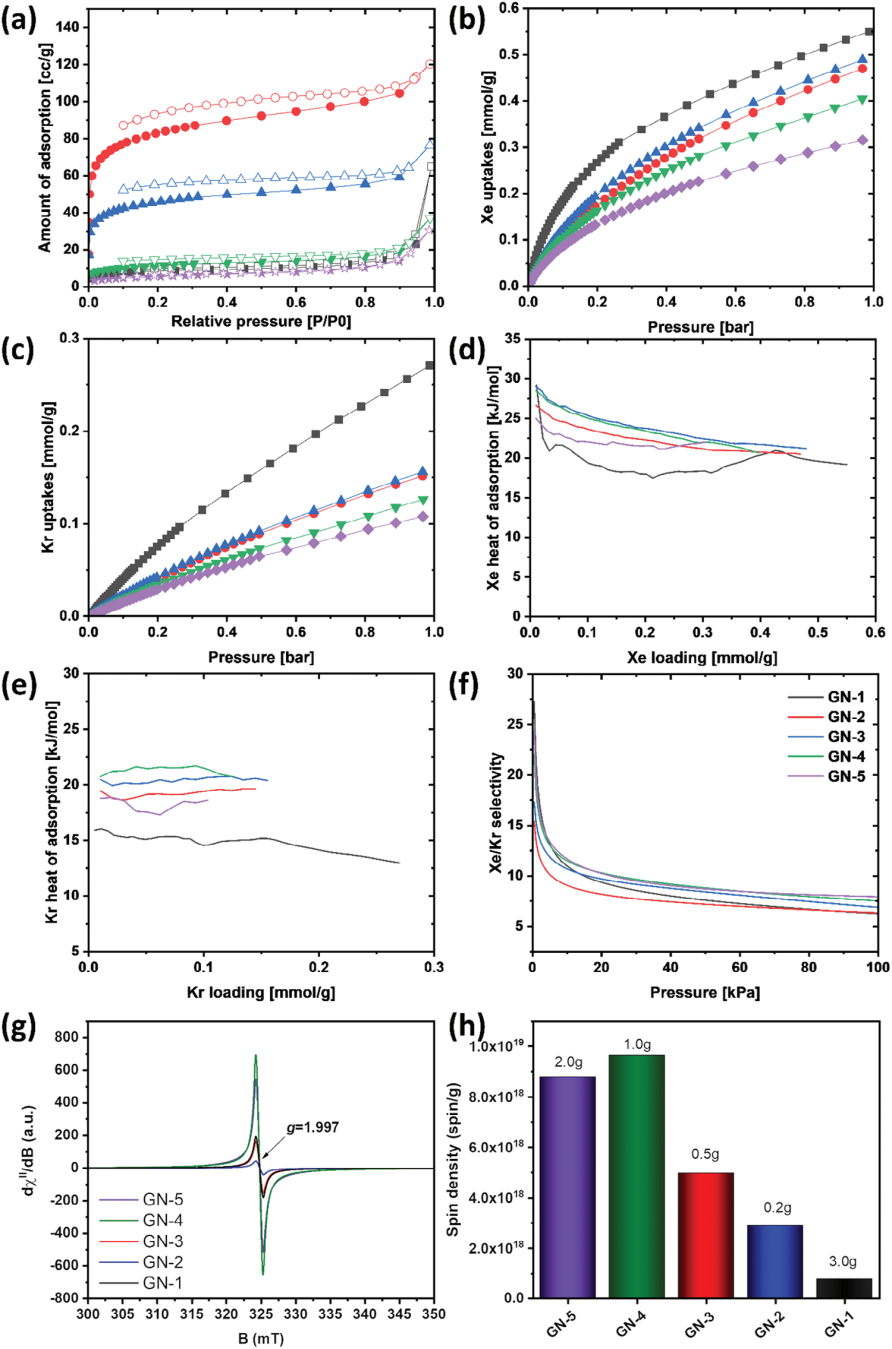
a) Nitrogen adsorption‐desorption isotherms measured at 77 K, b) Xe uptake measured at 298 K up to 1 bar, c) Kr uptake measured at 298 K up to 1 bar. d) The heat of adsorption (Qst) of (d) Xe and e) Kr. f) The IAST‐predicted Xe/Kr selectivity under Xe and Kr mixture (20%:80%) of GN samples (GN‐1 black, GN‐2 red, GN‐3 blue, GN‐4 green and GN‐5 purple color lines). g) X‐band CW EPR traces of nitrogen‐doped graphene (GNs) materials recorded at 85 K. h) The experimentally determined spin concentration (spin per g) within the series of studied materials together with the amount of sodium azide that was used in the synthesis of the sample.

We have measured nitrogen adsorption–desorption isotherms for all nitrogen‐doped graphenic carbon samples at 77 K. The Brunauer‐Emmett‐Teller (BET) surface area and total pore volume of the resultant nitrogen‐doped graphene samples, labeled as GN‐1, GN‐2, GN‐3, GN‐4, and GN‐5, are 32, 311, 171, 41, and 19 m^2^ g^−1^, and 0.040, 0.176, 0.107, 0.045, and 0.036 cm^3^ g^−1^, respectively, as shown in Figure S8a (Supporting Information). The resulting adsorption isotherms show a typical type‐IV isotherm (H1 type broad hysteresis loop) at higher relative pressure (P/P0 >4) (**Figure** **3**a). This behavior indicates the capillary condensation of nitrogen within the mesopores, attributed to the defects arising from vacancies and holes in the aromatic lattice, coupled with organized interlayer “nanochannels” and nitrogen functional groups. In order to obtain more information about the nature of the spin containing defects in the structure of the GNs materials, we performed a series of Electron Paramagnetic Resonance (EPR) experiments. All GNs samples exhibit the same isotropic EPR fingerprints, resulting in a sharp resonant line at *B* = 325 mT that gives *g* = 1.997 (Figure 3g). Furthermore, we did not observe any indication of a high‐spin state (e.g., *S* > 1/2), thus no indication of dipolar components, and zero‐field splitting terms. Moreover, the EPR spectra did not show any detectable interaction with the ^14^N (*I* = 1) atoms, indicative of the exclusive localization of spin active sites at the carbon centers. Although qualitatively, we did not observe any variations in the spectra, the different intensities of the EPR lines within this series of samples are evident. Taking into account that experiments were performed under identical experimental and resonance conditions, as well as was kept the same filling factor in all samples, then such differences arise from the different number of spin‐activated sites (defects) in the structure of each GN material, namely in their different spin densities. This led us to calculate the spin density, which can be directly related to the effective concentration of the spin defects in the samples; the materials exhibited a large range of spin concentration values, being GN‐1 (7.7 × 10^17^ spin g^−1^) encoding the smallest concentration and GN‐4 encompassing the largest (9.6 × 10^18^ spin g^−1^). Such differences can potentially translate into significant alteration of the system activity in response to the extent of accumulation of the electron spin charges (Figure 3h).

Using the density functional theory (DFT), we evaluated pore size distribution from the nitrogen isotherms at 77 K (Figure S9, Supporting Information). With the progressive infusion of a greater quantity of the nitrogen source, the pore dimensions expanded owing to the proliferation of defects within the graphene layer, consequently leading to a diminished BET surface area and overall pore volume. GN‐2 and GN‐3 showcase micropores spanning 10–16 Å, whereas GN‐1 shows micropores measuring 17 Å. In contrast, GN‐4 and GN‐5 feature larger mesopores accompanied by decreased BET surface areas. These unique properties suggest potential utility in noble gas storage and separation applications under low‐pressure conditions.

The individual single‐component Xe and Kr adsorption isotherms for GN samples were recorded at varying temperatures 273 K (Figure S10, Supporting Information) and 298 K (Figure 3b,c) and pressures up to 1 bar. The resulting samples analyzed for Xe and Kr adsorption consistently exhibited an Xe‐selective trend, given that Xe has a higher polarizability than Kr (40.4 × 10^−25^ cm^3^ for Xe and 24.8 × 10^−25^ cm^3^ for Kr).^[^ ^51^, ^52^ ^]^ The order of Xe and Kr uptakes of GN3 samples at 298 K and 1 bar were as follows: GN‐1 > GN‐2 > GN‐3 > GN‐4 > GN‐5 (Figure 3b,c). Interestingly, GN‐1 exhibits remarkable noble gas adsorption compared to other resulting materials, attributed to its high nitrogen content despite having a low BET surface area. Figure S8b (Supporting Information) illustrates the correlation between BET surface area, nitrogen content, and Xe uptake at 1 bar. As the amount of azide used in the synthesis of GN‐2, GN‐3, and GN‐4 material increases, from 0.2 g (for GN‐2) to 1.0 g (for GN‐4), both the amount of nitrogen incorporated into the carbon matrix and the pore size increases, while, on the contrary, the active surface area decreases. At low azide concentrations, nitrogen preferentially occupies pyrrolic positions (Figure S6, Supporting Information) and only then incorporates into pyridinic positions with higher azide concentrations, as the pyridinic N configuration is more stable.^[^ ^50^ ^]^ However, from GN‐5 (2 g of NaN_3_) to GN‐1 (3 g, in DMF), sp^3^ functionalization begins to increase dramatically, resulting in a subsequent decrease in pore volume and spin density, as evident from a Raman comparison of all prepared materials (Figure S5, Supporting Information). N‐doping, involving an electron donor dopant, induces substantial lattice distortion, resulting in an asymmetric charge distribution that enhances adsorption affinity with noble gases. However, the simultaneous occurrence of structural defects in graphene with N‐doping introduces uncertainty regarding the primary factor contributing to the enhancement of noble gas adsorption, necessitating appropriate optimization. Certainly, introducing an excessive amount of N source, as seen in GN‐5, results in the formation of large mesopores (>250 Å), ultimately diminishing Xe adsorption uptake. Nevertheless, Xe adsorption gradually rises with increasing N content, and GN‐1 stands out for its particularly high Xe and Kr adsorption capacities, demonstrating the effectiveness of optimized N‐doping. Therefore, the remarkable noble gas adsorption observed in GN‐1 could be attributed to a unique combination of pore size, nitrogen concentration, and appropriate amount of sp^3^ functionalization. Additionally, nitrogen functional groups and defects have been observed to induce an asymmetrical distribution of charges on the carbon surface, further intensifying the attraction between the charges on the nitrogen‐doped carbon surface and the polarizable Xe atoms.^[^ ^53^ ^]^

To evaluate the host–guest interaction between GN samples and noble gases, the heat of adsorption (Qst) of Xe and Kr were calculated by the Clausius–Clapeyron equation derived from Xe and Kr adsorption isotherms with two different temperatures of each GN sample. The pristine GN‐1 exhibited the highest heat of adsorption (Qst) for Xe at low loading, whereas it showed the lowest Kr Qst throughout the entire loading range (Figure 3d,e). On the other hand, the GN‐3 and GN‐4 samples exhibited high Xe and Kr Qst, which is consistent with the order of Xe adsorption uptake at low pressure. The Qst of Xe and Kr results demonstrate that the impact of nitrogen doping on the graphene surface is more pronounced in Xe and Kr adsorption compared to the micropore generated by defects in the graphene layers. This discrepancy indicates a pronounced Xe/Kr selectivity attributed to GN's robust binding energy with Xe molecules and comparatively weaker binding energy with Kr molecules (Figure 3f). To assess the separation performance under the Xe and Kr mixture, Xe and Kr adsorption isotherms of GN samples were fitted by dual‐sites Langmuir–Freundlich equation and calculated the Xe/Kr selectivity based on ideal adsorbed solution theory (IAST) using the IAST++ program.^[^ ^54^ ^]^ As shown in Figure 3f, GN‐1 exhibited the highest Xe/Kr selectivity at low pressure, primarily due to its strong interaction with Xe molecules and weaker interaction with Kr molecules. Interestingly, the Xe/Kr selectivity at low pressure of nitrogen‐doped GN samples followed an increasing order of nitrogen content, indicating a direct correlation between nitrogen doping on the graphene surface and its impact on Xe/Kr separation selectivity. These findings suggest that the interlayer spacing nanochannels of graphene material play a pivotal role in determining Xe and Kr adsorption properties, while micropores resulting from defects in the graphene layer have a more peripheral role. Furthermore, nitrogen functionalization of the graphene layer holds the potential to enhance Xe/Kr separation selectivity.

**Figure 4:**
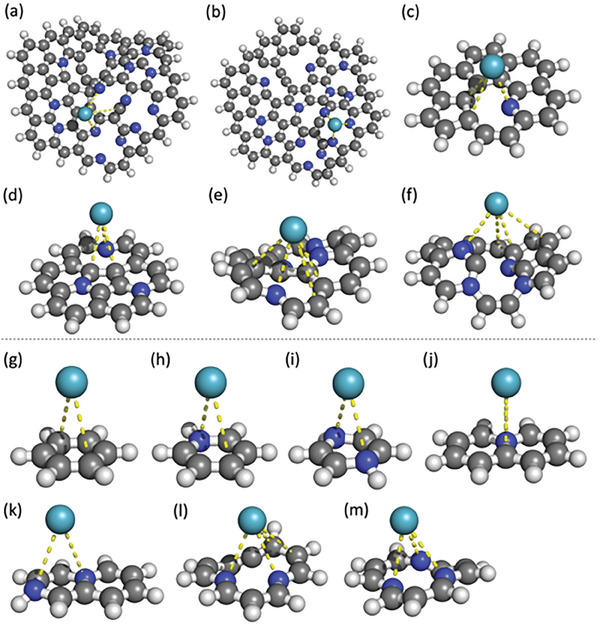
a–m) Kr/Xe@GN models. Carbon in gray, nitrogen in blue, hydrogen in white, and Kr/Xe in green.

The interactions of Kr and Xe noble gases with GN were also examined by first‐principle calculations (**Figure** **4**) using hybrid DFT functional with empirical dispersion correction D3 and couple‐cluster theory with single, double, and perturbative triple excitations (CCSD(T)), which is considered a gold standard of computational chemistry reaching thermodynamic accuracy. DFT based binding energies of Kr and Xe to GN range from −7.1 to −25.9 kJ mol^−1^ (Table S2, Supporting Information) and distances of noble gas (Kr/Xe) atoms to GN lie within 3.5–3.9 Å. The CCSD(T) calculations carried out using smaller and computationally feasible benzene models provide binding energies ranging from −5.2 to −9.4 kJ mol^−1^ (Table S3, Supporting Information). Both methods confirm a stronger interaction of Xe atoms to GN with respect to Kr. The values of binding energies as well as the bonding distances fit those values typical for noncovalent complexes. The SAPT analysis decomposing the interaction energy into physically relevant components including electrostatics, induction, dispersion, and exchange‐repulsion conducted on Kr@GN revealed the primary role of dispersion interaction (Table S4, Supporting Information), as expected for interaction of noble gas atoms. The calculations also revealed that the noble gas atoms tended to interact preferentially with nitrogen atoms than carbon atoms. The energy difference between Kr and Xe binding to GNs increased with increasing N atomic content in GN structure, especially when similar models were compared (Tables S2 and S3, Supporting Information), signifying the importance of nitrogen doping for the noble gas separation. The theoretical calculations are also in good agreement with experimentally derived enthalpies of Kr/Xe–GN adsorption (Table S5, Supporting Information), in addition, they provide deep physical‐chemical insights into the nature of underlying interactions.

In summary, we have synthesized nitrogen‐doped graphene materials achieving effective nitrogen incorporation, impressive interlayer spacing, and defects exploited for noble gas storage and separation. The resulting materials maintained abundant aromatic regions, undergoing significant nitrogen doping within the vacancies and holes of the aromatic lattice. The extent of such defective structures was unraveled by EPR spectroscopy. It was found that the diverse N‐doped materials exhibited a substantial variation in the total number of spin defects (spin density), hence the properties of these systems varied from strong paramagnetic to nearly diamagnetic. The organized interlayer nanochannel and N‐doping not only significantly contributed to noble gas adsorption despite defects within the 2D structure but also exhibited selectivity for Xe over Kr separation. N‐doped graphene demonstrated increasing Xe and Kr adsorption uptakes with the gradual increase in N content. This can be attributed to N‐doping with electron donor dopants inducing an asymmetric charge distribution, thereby enhancing the adsorption affinity with noble gases. Furthermore, defects resulting from N‐doping formed micropores, offering additional adsorption sites for noble gases. This assertion is supported by theoretical calculations employing symmetry‐adapted perturbation theory analysis. This emphasizes the importance of nitrogen functional groups, sp^3^ defects/functionalization, and interlayer nanochannels over the surface area in achieving enhanced selectivity. This study paves the way for exploring various 2D materials for noble gas storage and separation applications, opening new avenues for research in this field.

## Conflict of Interest

The authors declare no conflict of interest.

## Supporting information



Supporting Information

## Data Availability

The data that support the findings of this study are available from the corresponding author upon reasonable request or at the ZENODO repository (https://doi.org/10.5281/zenodo.13993402).
