# Supplementary material for: 2D Nitrogen‐Doped Graphene Materials for Noble Gas Separation
Source: Small. 2024 Nov 6;21(6):2408525. doi: 10.1002/smll.202408525 (PMC11817904; doi:10.1002/smll.202408525)
Supplement: Supplementary file 1 — Supporting Information [file SMLL-21-2408525-s001.docx]

Supporting Information

**Two-Dimensional Nitrogen-Doped Graphene Materials for Noble Gas Separation**

Veronika Šedajová^#^, Min-Bum Kim^#^, Rostislav Langer, Gobbilla Sai Kumar, Lili Liu, Zdeněk Baďura, James V. Haag, Giorgio Zoppellaro, Radek Zbořil, Praveen K Thallapally*, Kolleboyina Jayaramulu*, Michal Otyepka*

Dr. V. Šedajová, Dr. Z. Baďura, Dr. G. Zoppellaro, Prof. R. Zbořil, Prof. K. Jayaramulu and Prof. M. Otyepka

Regional Centre of Advanced Technologies and Materials, Czech Advanced Technology and Research Institute (CATRIN), Palacký University, Šlechtitelů 27, 783 71, Olomouc, Czech Republic.

Email: [michal.otyepka@upol.cz](mailto:michal.otyepka@upol.cz)

Email: [jayaramulu.kolleboyina@upol.cz](mailto:jayaramulu.kolleboyina@upol.cz)

Dr. MB. Kim, Dr. Lili Liu, Dr. J.V. Haag, Prof. P. K Thallapally

Pacific Northwest National Laboratory, Richland, Washington 99352, United States

Email: [Praveen.Thallaplly@pnnl.gov](mailto:Praveen.Thallaplly@pnnl.gov)

Prof. K. Jayaramulu, Dr. G.S. Kumar

Department of Chemistry, Indian Institute of Technology Jammu, Jammu and Kashmir 181221, India

Dr. Z. Baďura, Dr. G. Zoppellaro, Prof. R. Zbořil

Nanotechnology Centre, CEET, VŠB-Technical University of Ostrava, 17. listopadu 2172/15, Ostrava-Poruba 708 00, Czech Republic.

Prof. M. Otyepka, Dr. R. Langer

IT4Innovations, VŠB–Technical University of Ostrava, 17. listopadu 2172/15, 708 00 Ostrava-Poruba, Czech Republic

#Equally contributed to this work

**1. Experimental Section**

**1.1. Materials**

Graphite fluoride (>61 wt% F), NaN3 (BioXtra), Tamisolve (N-butylpyrrolidone, ≥99.5%) and N,N-Dimethylformamide (DMF, ≥98%) were procured from Sigma-Aldrich. Acetone and ethanol (absolute) were obtained from Penta, Czech Republic. All chemicals were used without further purification. Ultrapure water was utilized for preparing aqueous solutions. Membra-cell® MD44 dialysis membranes were employed for dialysis. Ultrapure water (18 MΩ cm–1) was employed for washings and dialysis.

**1.2 Synthesis of nitrogen doped graphene (GN) samples**

GN materials were prepared using fluorographene chemistry according to the published protocol (for GN-1),^[1]^ which was modified for other GNs, i.e., GN-2, GN-3, GN-4, and GN-5, as described below. Particularly, graphite fluoride (1 g) was dispersed in 60 ml of DMF and stirred using a Teflon-coated magnetic stirrer at 500 rpm for 3 days in a glass flask. Following this, the mixture underwent 4 hours of sonication (Bandelin Sonorex, DT255H type, frequency 35 kHz, effective power 160 W), followed by overnight stirring. On the subsequent day, 3 g of NaN_3_ was added, and the dispersion mixture was heated at 130 ºC for 72 h with a condenser, stirring at 800 rpm in an oil bath. After completing the reaction, the mixture cooled, and washing was conducted sequentially with DMF (or Tamisolve for other GNs, such as GN-2, GN-3, GN-4, and GN-5), acetone, ethanol, hot water (80 ^o^C), water, and acidified water (3% HCl solution). Product separation (GN-1) from solvents was achieved through centrifugation (Sigma 4-16K) at 13,000 rcf. Subsequently, washing with water continued until the material ceased precipitating with the centrifuge. To vary the nitrogen percentage, textural parameters, and environmental support, the toxic solvent DMF was replaced with the eco-solvent Tamisolve. The quantity of the N-source (sodium azide) was then adjusted to achieve different N-dopings. Specifically, for 1 g of graphite fluoride (initial material), we added 0.2 g, 0.5 g, 1 g, and 2 g to the mixture, resulting in products named GN-2, GN-3, GN-4 and GN-5, respectively.

**Table S1** shows synthetic details of different compositions of GN samples.

| Sample Code | Graphite fluoride  (g) | Sodium Azide  (g) | DMF  (ml) | Tamisolve  (ml) |
| --- | --- | --- | --- | --- |
| **GN-1** | 1 | 3.0 | 60 | - |
| **GN-2** | 1 | 0.2 | - | 60 |
| **GN-3** | 1 | 0.5 | - | 60 |
| **GN-4** | 1 | 1.0 | - | 60 |
| **GN-5** | 1 | 2.0 | - | 60 |

**1.3 Characterization Techniques**

XRD measurements were performed using an X’Pert PRO MPD diffractometer (PANalytical) in the Bragg–Brentano geometry equipped with a Co X-ray tube. Samples were placed on a zero-background Si slide, gently pressed, and scanned with a step size of 0.0334°, and the 2θ range of 5°–120° was used to record the pattern. Infra-red spectra were acquired using an iS5 FTIR spectrometer (Thermo Nicolet) with the Smart Orbit ATR accessory featuring a ZnSe crystal. A drop of sample dispersion in ethanol or water was placed on a ZnSe crystal and left to dry, forming a film in ambient conditions, and spectra were recorded by summing 50 scans. Nitrogen gas flowed through the ATR accessory during background and sample measurements, and ATR and baseline correction were applied for spectrum processing. X-ray photoelectron spectroscopy (XPS) was conducted using a PHI VersaProbe II (Physical Electronics) and Nexsa G2 (Thermo Fisher) spectrometers with an Al Kα source. Binding energies were referenced to the C1s core level of the C-C bond at the nominal value of 284.8 eV. Data were evaluated and deconvoluted with the MultiPak (Ulvac-PHI, Inc.) software package and Avantage software (Thermo Fisher). Transmission electron microscopy (TEM) images were obtained using a JEOL 2100 TEM with an emission gun of LaB_6_ type operating at 160 kV. High-resolution TEM images were obtained using an FEI Titan electron microscope operating at 80 kV. Scanning electron microscopy (SEM) was performed using a Hitachi SU6600 instrument with an accelerating voltage of 5 kV. For these analyses, a small droplet of a material dispersion in ultrapure water (concentration approximately 0.1 mg/ml) was placed on a carbon-coated copper grid and left to dry. Raman spectra were obtained on a DXR Raman microscope using the 633 nm excitation line diode laser. The nitrogen adsorption/desorption isotherms at 77 K were measured by using 3Flex instrument (Micromeritics Instruments, Norcross, GA, USA). Before the nitrogen adsorption/desorption experiments, the GN samples were degassed at 100 ºC for 16 h under vacuum. The BET surface areas of the samples were calculated using BET model from nitrogen isotherms at 77 K. The single-component of Xe and Kr adsorption isotherms were conducted on samples with activated at 100 ºC for 16 h under vacuum and measured by a 3Flex instrument at varying temperatures (273 K and 298 K). A target temperature was maintained using a water circulation system. EPR spectra were collected on X-band (∼9.14–9.17 GHz) spectrometer JEOL JES-X-320 equipped by variable He temperature set-up ES-CT470 apparatus. The quality factor (Q) was kept above 6500 for all measurements to make the spectra comparable. The accuracy of the *g*-values was determined by comparison with a Mn^2+^/MgO standard (JEOL standard). The microwave power was set to 1.0 mW to avoid power saturation effects, only sample GN-1 was measured at 5 mW. A modulation width of 1.00 mT and a modulation frequency of 100 kHz were used. All EPR spectra were collected with a time constant of 30 ms and a sweep time of 4 minutes. As a sample holder were used high purity quartz tubes (Suprasil, Wilmad, ≤0.5 OD) and for standard powder experiments (Figure 3g) 5 mg of samples were transferred into the tube and which was subsequently purged by nitrogen gas for 10 minutes. The experimental temperature for powder spectra was set to 85 K. For spin density experiments, powder samples were placed in polypropylene VSM (P125E) holders and the sealed capsules were inserted into the quartz EPR tube. In this way, the same tube placed at the same height inside the CW-resonator cavity could be used for all measurements. The measurements were performed at 160 K, keeping the experimental parameters the same for all experiments performed. The filling factors were kept as close to the same as possible, while a ruler was used to check the final heights after loading. The amount of sample powders loaded in P125E were (i) 6.76 mg for GN-1, (ii) 9.21 mg for GN-2, (iii) 7.94 mg for GN-3, (iv) 4.96 mg for GN-4, and (v) 6.86 mg for GN-5. To evaluate the spin density of a paramagnetic material (ST) was used the CuSO_4_ × 5H_2_O (99.999%, CAS Number: 7758-99-8) as S =1/2 spin reference placed in the same VSM holder with 6.45 mg loading. The double integrated EPR signal intensities (Ds) of sample and standard must be divided by the square root of the applied microwave power used during signal acquisition, ∫∫EPRint/√P. Sample and standard must be recorded using the same experimental conditions (T(K), gain, scan field rate) and then Equation (1) can be applied in the S_T_ evaluation. Below is given the procedure to calculate the spin density.

| $\text{S}_{\text{T}}\text{= }\left( \text{ α × }\frac{\int\int\text{EPRint }\left( \text{GN} \right)\text{ }}{\text{ }\int\int\text{EPRint }\left( \text{CuS}\text{O}_{\text{4}}\text{ } \right)}\text{ } \right) \times\frac{m_{\text{CuS}\text{O}_{\text{4}}}}{m_{\text{GN}}} \times N_{A \left( \text{CuS}\text{O}_{\text{4}} \right)}$ | (1) |
| --- | --- |

The terms given in Equation (1), represent: N_A_ = Avogadro number, 6.02214×1023, and (i) N_A_ (CuSO_4_) = 2.41 ×10^21^ (per molecule), or 9.48×10^21^ per Cu^2+^ cation, (ii) *g*_CuSO4_ = grams of copper sulphate pentahydrate standard used, (iii) *g* sample material, (iv) α = *g* values ratio, *g*_avg_ (CuSO_4_) /g_avg_ sample with gavg (CuSO_4_ × 5 H_2_O) = 2.172. Figure S11 shows the X-band of solid CuSO_4_ in the 303-143 K temperature range, and Fig. S12 the calculated spin densities in the same range, showing its paramagnetic behaviour. The g_avg_ value for the sample must be determined from the recorded EPR spectrum (g_avg_ = [g_x_ + g_y_ + g_z_ /3]).

**1.4 Computational details**

The first principle calculations including geometry optimizations, evaluation of the binding energies and Kr/Xe–GN distances were employed by using Gaussian16 software^[2]^ in three setups: i) B3LYP functional^[3]^ with D3 empirical dispersion^[4]^ and def2-SVP basis set,^[5,6]^ ii) ωb97xd^[7]^ with def2-SVP basis set,^[5,6]^ and iii) CCSD(T)^[8,9]^ with def2-TZVP basis set.^[5,6]^ The GN models were chosen based on a previous study by Šedajová *et al.*^[1]^ Moreover, we used smaller GN models of N-doped benzene, anthracene, phenalene, and coronene to properly examine the binding preferences, and to use more demanding coupled cluster method. Here, we pre-optimized models with B3LYP+D3 functional and run single point CCSD(T) calculations.

The strength of the interaction of Kr/Xe with GN was evaluated by binding energies as $E_{bind}=(E_{GN+X}- E_{GN}-E_{X})$, where $E_{GN+X}$, $E_{GN}$, and $E_{X}$, denote total energies of whole GN + noble gas system, GN model, and noble gas (X = Kr, Xe), respectively. The enthalpies, $\Delta H$, of the Kr/Xe–GN interaction for temperature of 298.15 K and pressure 1 atm was evaluated at the B3LYP/def2-SVP level of theory as $\Delta H=\Delta E+ \Delta ZPVE+{\Delta E}_{corr}+{\Delta H}_{corr}$, where $\Delta E$ is the electronic energy, $\Delta ZPVE$ is the zero-point vibrational energy, ${\Delta E}_{corr}$ is the thermal energy correction, and ${\Delta H}_{corr}$ is the thermal correction to enthalpy. The energy decomposition was performed by the Symmetry-Adapted Perturbation Theory (SAPT)^[10,11]^ at the basis aug-cc-pVDZ and SAPT0 level as implemented in PSI4 package.^[12]^

**References**

[1] V. Šedajová, A. Bakandritsos, P. Błoński, M. Medveď, R. Langer, D. Zaoralová, J. Ugolotti, J. Dzíbelová, P. Jakubec, V. Kupka, M. Otyepka, *Energy Environ. Sci.* **2022**, DOI 10.1039/D1EE02234B.

[2] Gaussian 16, Revision C.01, M. J. Frisch, G. W. Trucks, H. B. Schlegel, G. E. Scuseria, M. A. Robb, J. R. Cheeseman, G. Scalmani, V. Barone, G. A. Petersson, H. Nakatsuji, X. Li, M. Caricato, A. V. Marenich, J. Bloino, B. G. Janesko, R. Gomperts, B. Mennucci, H. P. Hratchian, J. V. Ortiz, A. F. Izmaylov, J. L. Sonnenberg, D. Williams-Young, F. Ding, F. Lipparini, F. Egidi, J. Goings, B. Peng, A. Petrone, T. Henderson, D. Ranasinghe, V. G. Zakrzewski, J. Gao, N. Rega, G. Zheng, W. Liang, M. Hada, M. Ehara, K. Toyota, R. Fukuda, J. Hasegawa, M. Ishida, T. Nakajima, Y. Honda, O. Kitao, H. Nakai, T. Vreven, K. Throssell, J. A. Montgomery, Jr., J. E. Peralta, F. Ogliaro, M. J. Bearpark, J. J. Heyd, E. N. Brothers, K. N. Kudin, V. N. Staroverov, T. A. Keith, R. Kobayashi, J. Normand, K. Raghavachari, A. P. Rendell, J. C. Burant, S. S. Iyengar, J. Tomasi, M. Cossi, J. M. Millam, M. Klene, C. Adamo, R. Cammi, J. W. Ochterski, R. L. Martin, K. Morokuma, O. Farkas, J. B. Foresman, and D. J. Fox, Gaussian, Inc., Wallingford CT, **2016**.

[3] A. D. Becke, *J. Chem. Phys.* **1993**, *98*, 5648.

[4] S. Grimme, J. Antony, S. Ehrlich, H. Krieg, *J. Chem. Phys.* **2010**, *132*, 154104.

[5] F. Weigend, R. Ahlrichs, *Phys. Chem. Chem. Phys.* **2005**, *7*, 3297.

[6] A. Schäfer, C. Huber, R. Ahlrichs, *J. Chem. Phys.* **1994**, *100*, 5829.

[7] J.-D. Chai, M. Head-Gordon, *Phys. Chem. Chem. Phys.* **2008**, *10*, 6615.

[8] J. Čížek, *J. Chem. Phys.* **1966**, *45*, 4256.

[9] K. Raghavachari, G. W. Trucks, J. A. Pople, M. Head-Gordon, *Chem. Phys. Lett.* **1989**, *157*, 479.

[10] B. Jeziorski, R. Moszynski, K. Szalewicz, *Chem. Rev.* **1994**, *94*, 1887.

[11] K. Szalewicz, *WIREs Comput. Mol. Sci.* **2012**, *2*, 254.

[12] D. G. A. Smith, L. A. Burns, A. C. Simmonett, R. M. Parrish, M. C. Schieber, R. Galvelis, P. Kraus, H. Kruse, R. Di Remigio, A. Alenaizan, A. M. James, S. Lehtola, J. P. Misiewicz, M. Scheurer, R. A. Shaw, J. B. Schriber, Y. Xie, Z. L. Glick, D. A. Sirianni, J. S. O’Brien, J. M. Waldrop, A. Kumar, E. G. Hohenstein, B. P. Pritchard, B. R. Brooks, H. F. Schaefer III, A. Yu. Sokolov, K. Patkowski, A. E. DePrince III, U. Bozkaya, R. A. King, F. A. Evangelista, J. M. Turney, T. D. Crawford, C. D. Sherrill, *J. Chem. Phys.* **2020**, *152*, 184108.


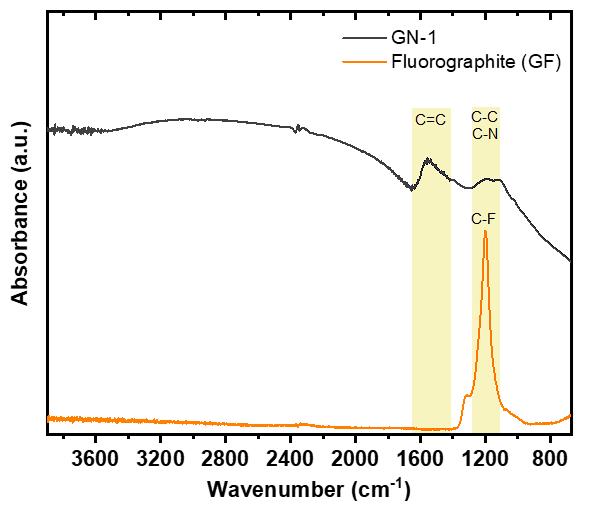


**Figure S1**. Fourier-transform infrared spectroscopy (FT-IR) spectra of pure FG (red line) and GN-1 (green line) samples.


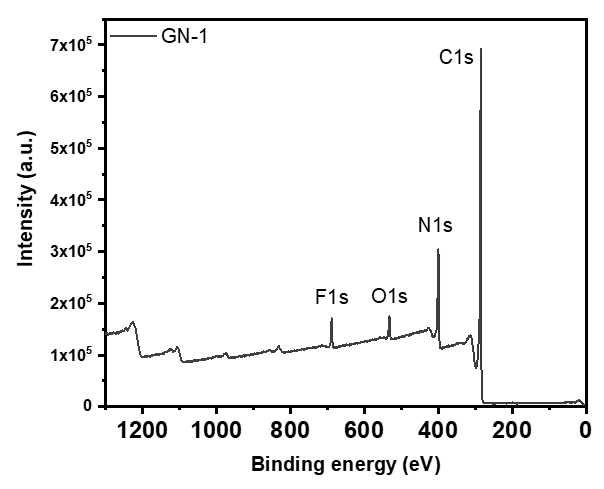


**Figure S2**. Survey spectrum from XPS analysis of GN-1.


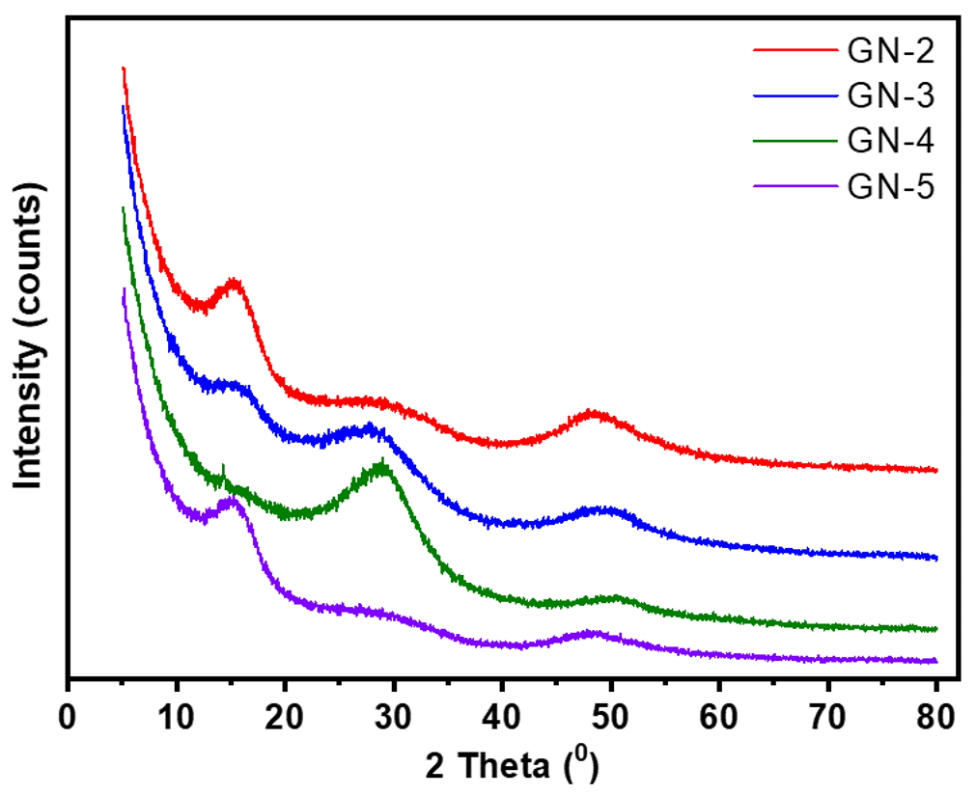


**Figure S3**. Powder XRD patterns of patterns of GN samples.

**
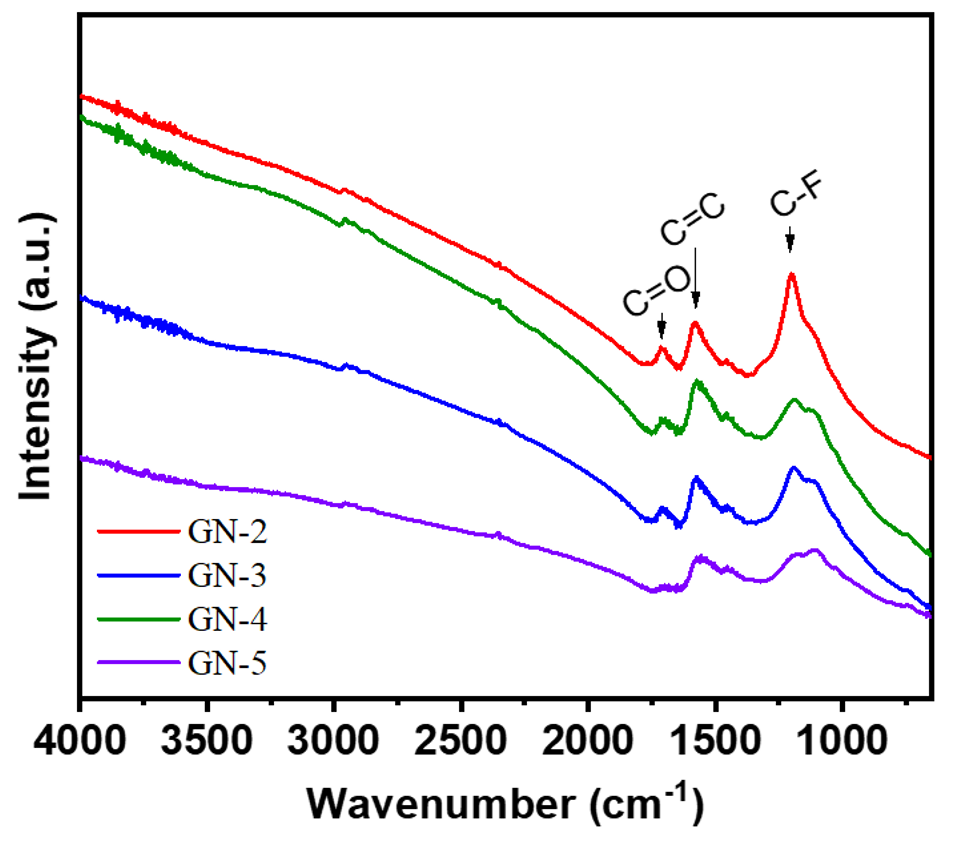
**

**Figure S4**. FT-IR patterns of patterns of resultant GN samples.


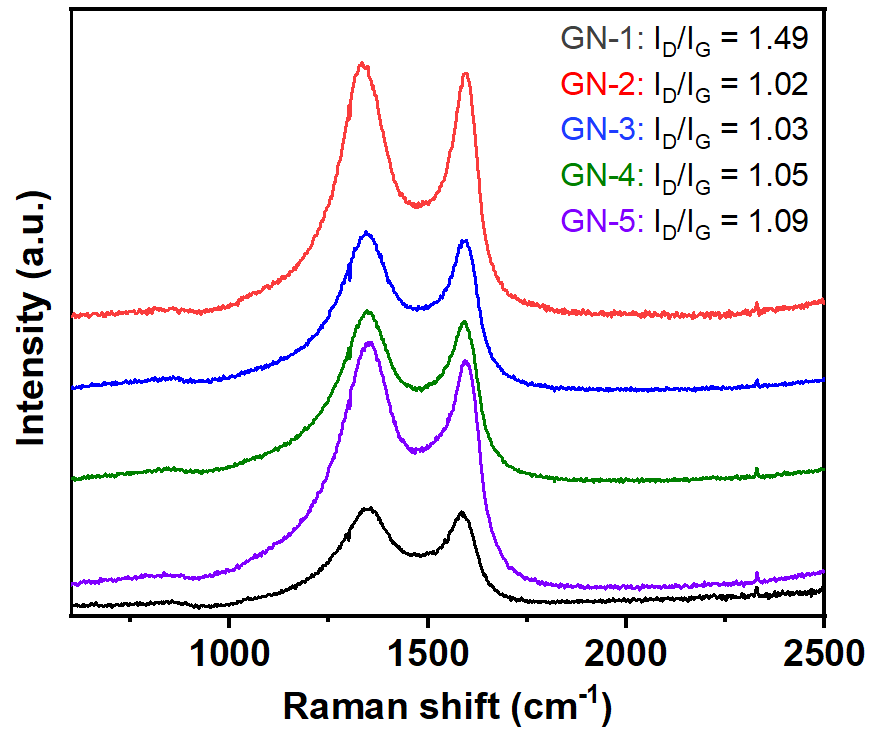


**Figure S5**. Raman patterns of GN samples with corresponding I_D_/I_G_ ratios.

**
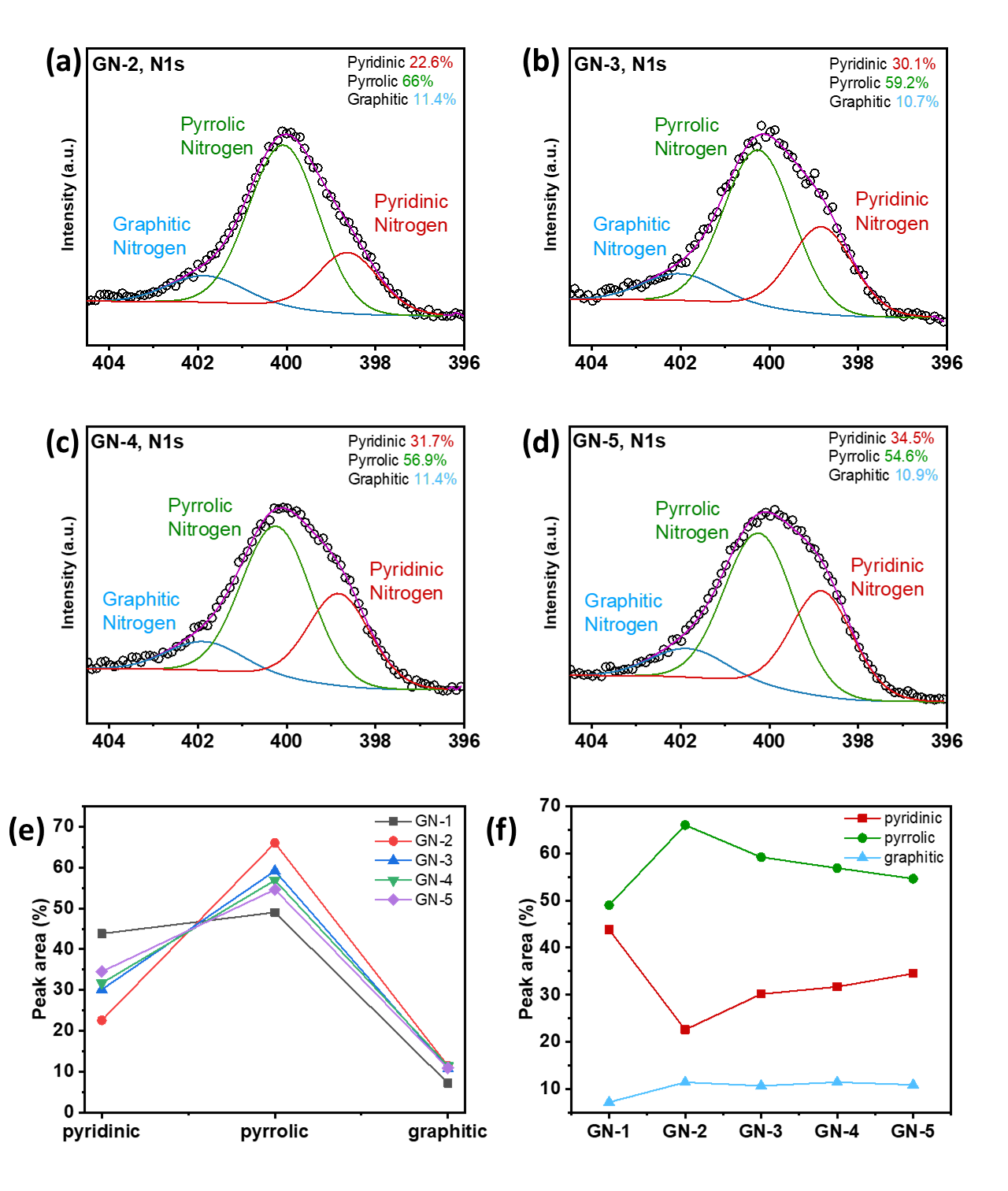
**

**Figure S6**. (a)-(d) HR-XPS of N1s regions from GN-2, GN-3, GN-4 and GN-5, respectively. (e),(f) Trends in the evolution of the different types of nitrogen in the GN samples.

**Description:**

The phase purity, structural, and morphology characterization through powder XRD, Raman, FT-IR, and SEM analyses of resultant GN samples. The PXRD pattern displayed distinct peaks around 30° and 1°, corresponding to (002) and (100) reflections, respectively. In FT-IR, bands at 1580 and 1210 cm^−1^ were attributed to aromatic carbon sp^2^, while 1400 cm^−1^ was assigned to pyridinic nitrogen substitution. The Raman spectra of the GNs showed prominent D and G band peaks. The I_D_/I_G_ band ratios indicate the presence of a significant number of non-heat-susceptible sp^3^ carbons and non-repairable defects, as the D band is associated with sp^2^ rings with adjacent sp^3^ defects, while G band relates to conjugated sp^2^ islands. Interestingly, the ratio increases with increasing N content in GNs prepared from Tamisolve solvent. The increasing concentration of azide in samples GN-2 and GN-3 results in the exclusive incorporation of nitrogen, leading to an increase in pore volume and surface area (Fig. 3) and defect concentration (Fig. 4). The sp^3^ functionalization and the formation of tetrahedral sp^3^ C-C bonds starts to be significant for the GN-4 and GN-5 samples, with the highest values for the GN-1 sample (DMF plays a crucial role in the tetrahedral bond formation process). As a result, both pore volume and surface area gradually decrease and the spindensity saturates. Comparative Raman spectroscopy for all samples confirmed this trend with an increasing I_D_/I_G_ ratio, peaking for the sample prepared with 3 g azide in DMF (GN-1). In addition, nitrogen HR-XPS showed an increasing amount of pyridinic nitrogen in the samples, again peaking at sample GN-1, further supporting our hypothesis.

X-ray photoelectron spectroscopy (XPS) of GNs revealed peaks corresponding to carbon, nitrogen, oxygen, and fluorine atoms, with atomic percentages detailed in Table 1.

Scanning electron microscopy (HRTEM) analysis of GN-1 at various magnifications showed randomly organized nanosheets. In summary, we synthesized nitrogen-doped graphitic carbon materials (GN-2, GN-3, GN-4, and GN-5) featuring nitrogen-based defects and functional groups on the graphitic plane.


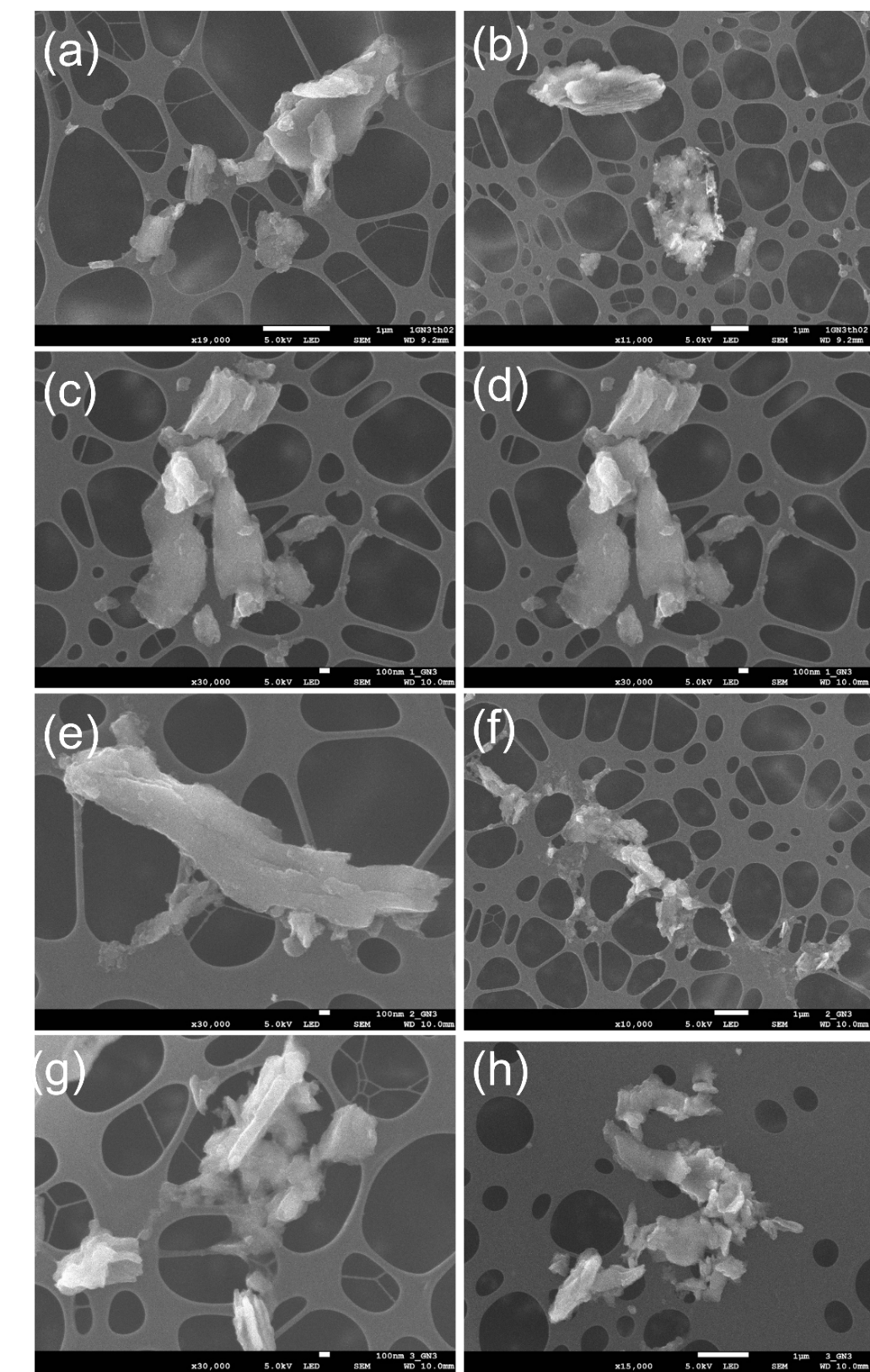


**Figure S7**. SEM images of GN samples as (a-b) GN-2; (c-d) GN-3; (e-f) GN-4 and (g-h) GN-5 samples


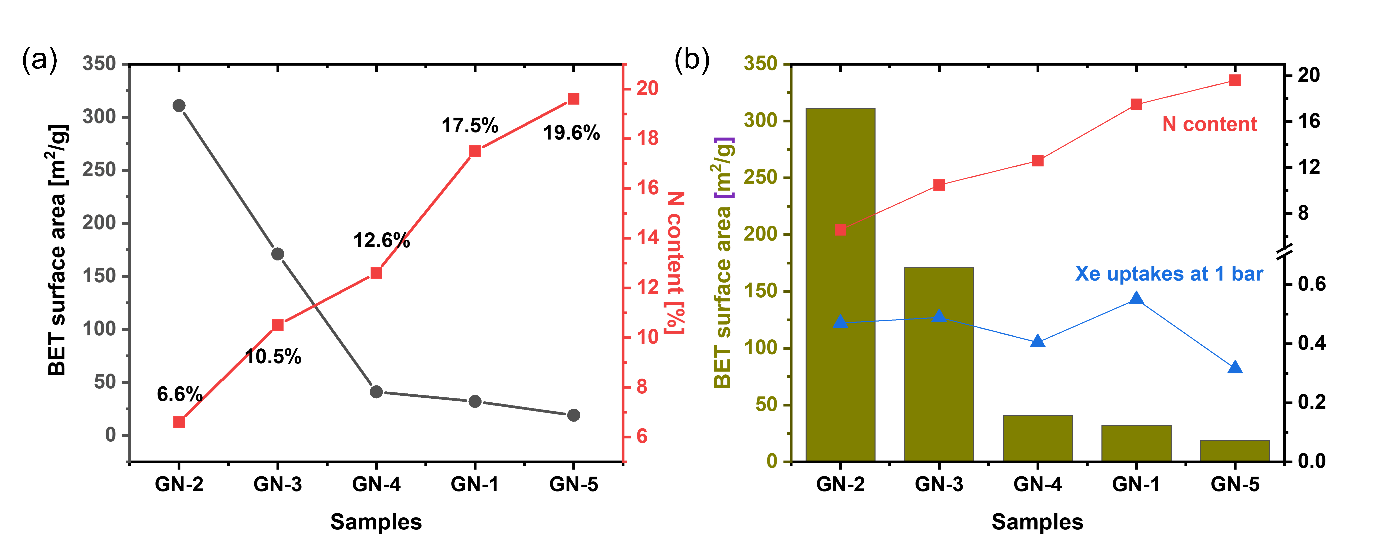


**Figure S8**. (a) Depicts the correlation between the nitrogen amount and the surface area of the NG samples; (b) illustrates the Xe uptake at 1 bar.


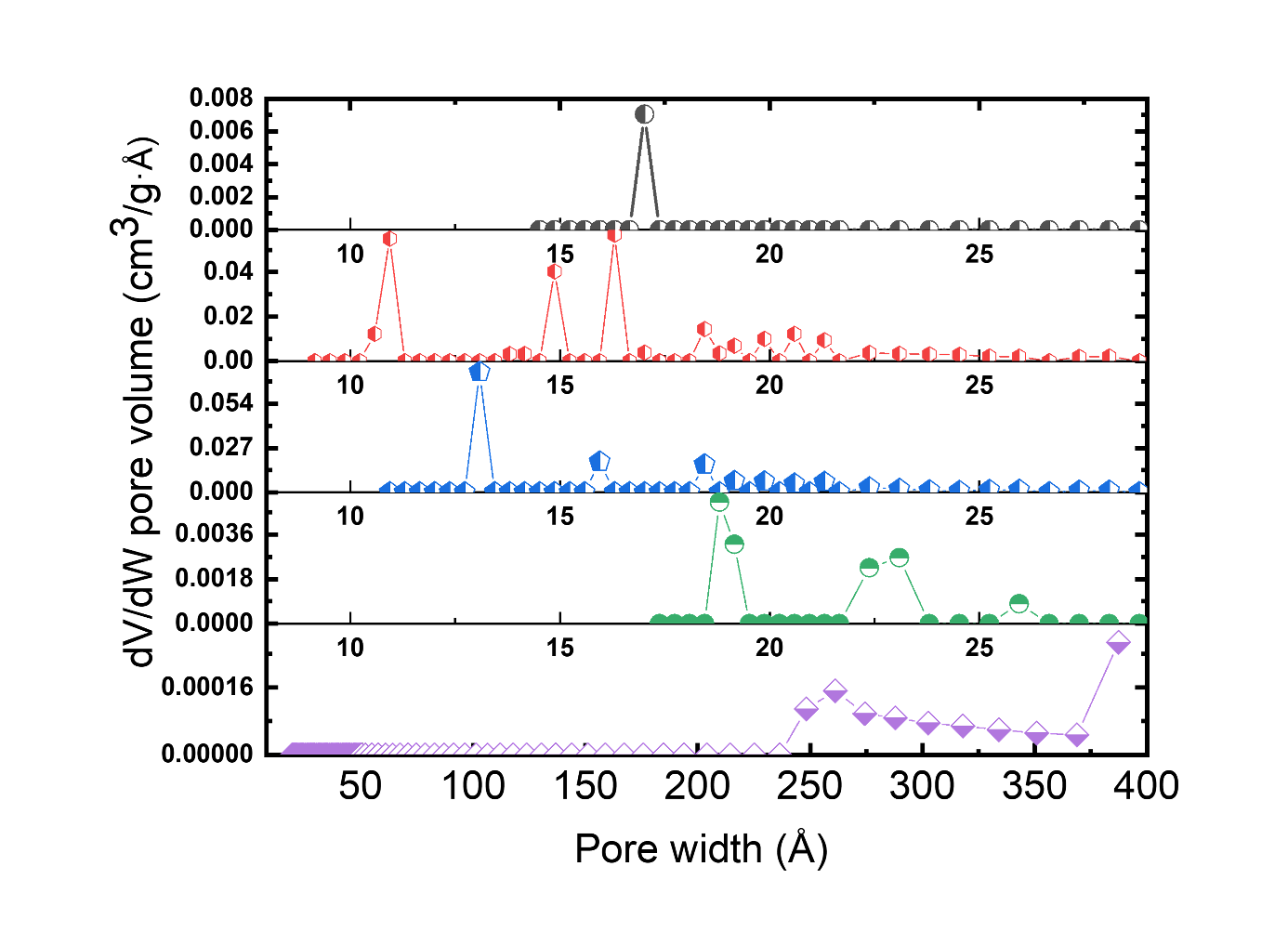


**Figure S9**. Pore size distribution measured nitrogen adsorption isotherm measured at 77 K through DFT method of resultant GN samples, where **(**GN-1 black; GN-2 red, GN-3 blue, GN-4 green, and GN-5 purple colour lines)


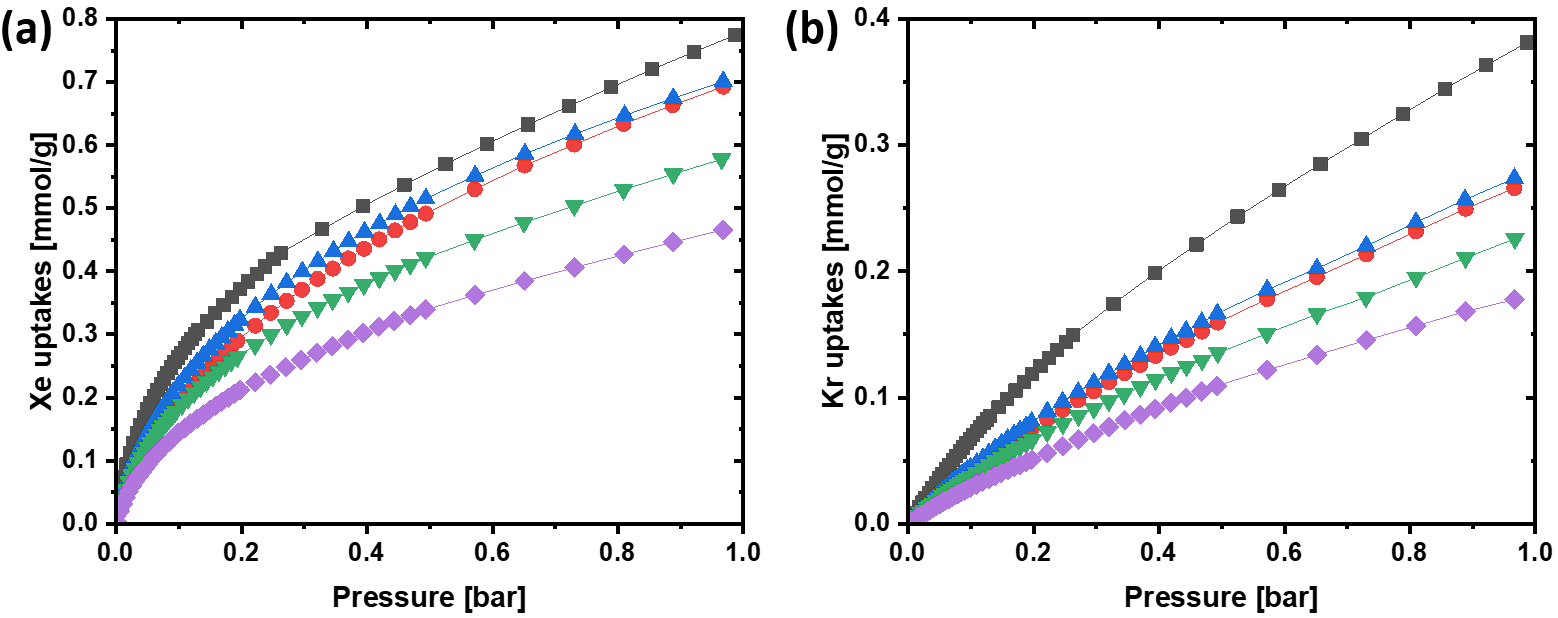


**Figure S10**. (a) Xe (b) Kr update measured at 273 K up to 1 bar of all GN samples (**(**GN-1 black; GN-2 red, GN-3 blue, GN-4 green, and GN-5 purple colour lines).


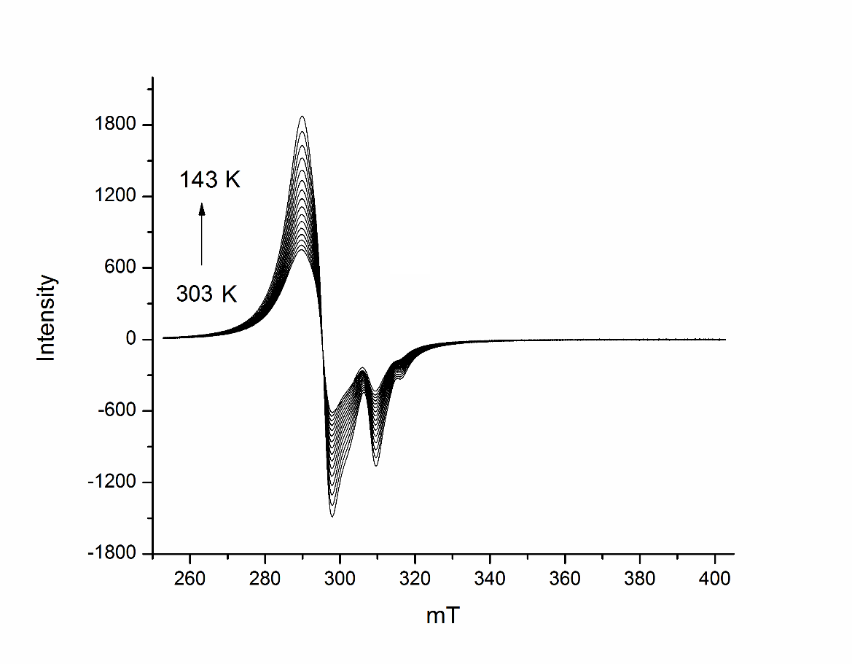


**Figure S11**. X-band (9.08 GHz) EPR spectrum of solid CuSO_4_ × 5 H_2_O spin (S =1/2) standard. The applied microwave power was set to 0.01 mW. The scan time was set to 4 min and the modulation width set to 1.0 mT.


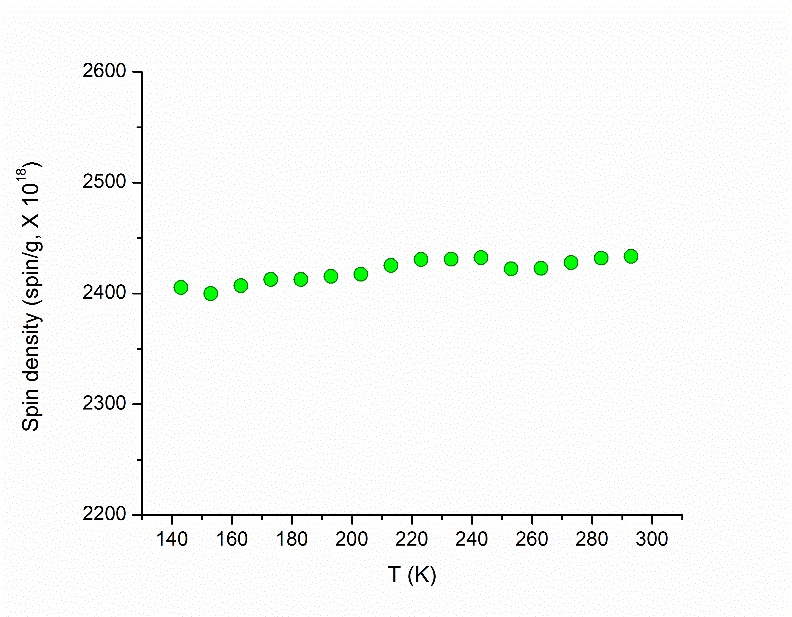


**Figure S12**. The CuSO_4_ × 5 H_2_O spin density vs Temperature obtained experimentally from X-band EPR experiments and analysis in the range 303-143 K.

**Table S2** Binding energies (kJ/mol) of Kr/Xe@GN obtained by using B3LYP+D3/def2-SVP and ωb97xd/def2-SVP methods. Structures correspond to Figure 4a-f. *∆E_bind_* denotes energy difference between the binding energies of Kr and Xe to GN.

| **structure** | **B3LYP+D3/def2-SVP** | | | | **ωb97xd/def2-SVP** | | | |
| --- | --- | --- | --- | --- | --- | --- | --- | --- |
|  | **Kr** | **Xe** | ***∆E_bind_*** | **N at.%** | **Kr** | **Xe** | ***∆E_bind_*** | **N at.%** |
| **a** | -23.39 | -25.90 | 2.51 | 12.17 | -23.35 | -25.31 | 1.97 | 12.17 |
| **b** | -14.77 | -18.49 | 3.72 | 12.17 | -12.47 | -17.03 | 4.56 | 12.17 |
| **c** | -16.82 | -17.95 | 1.13 | 2.78 | -14.90 | -17.82 | 2.93 | 2.78 |
| **d** | -15.52 | -17.49 | 1.97 | 8.33 | -14.14 | -17.66 | 3.51 | 8.33 |
| **e** | -15.27 | -16.11 | 0.84 | 11.43 | -7.07 | -15.94 | 8.87 | 11.43 |
| **f** | -15.61 | -20.92 | 5.31 | 14.29 | -14.39 | -19.54 | 5.15 | 14.29 |

**Table S3** Binding energies (kJ/mol) of Kr/Xe@GN obtained by using B3LYP+D3/def2-SVP and CCSD(T)/def2-TZVP methods. Structures correspond to Figure 4g-m. *∆E_bind_* denotes energy difference between the binding energies of Kr and Xe to GN.

| **structure** | **B3LYP+D3/def2-SVP** | | | | **CCSD(T)/def2-TZVP** | | | |
| --- | --- | --- | --- | --- | --- | --- | --- | --- |
|  | **Kr** | **Xe** | ***∆E_bind_*** | **N at.%** | **Kr** | **Xe** | ***∆E_bind_*** | **N at.%** |
| **g** | -8.74 | -9.33 | 0.59 | 0.00 | -5.23 | -7.11 | 1.88 | 0.00 |
| **h** | -9.29 | -10.54 | 1.26 | 7.69 | -6.02 | -8.08 | 2.05 | 7.69 |
| **i** | -9.62 | -12.26 | 2.64 | 15.38 | -6.95 | -9.41 | 2.47 | 15.38 |
| **j** | -12.34 | -12.72 | 0.38 | 5.26 | - | - | - | - |
| **k** | -12.43 | -12.97 | 0.54 | 10.53 | - | - | - | - |
| **l** | -14.77 | -16.61 | 1.84 | 9.09 | - | - | - | - |
| **m** | -14.85 | -18.03 | 3.18 | 13.64 | - | - | - | - |

**Table S4** SAPT analysis (kJ/mol) of Kr@GN. Structures correspond to Figure 4g-m.

| **Electrostatics** | **Exchange** | **Induction** | **Dispersion** |
| --- | --- | --- | --- |
| **structure g** | | | |
| -5.40 | 13.84 | -1.13 | -14.02 |
| **structure i** | | | |
| -5.65 | 14.06 | -1.34 | -14.92 |
| **structure k** | | | |
| -5.97 | 14.78 | -0.98 | -18.48 |

**Table S5** Enthalpies (kJ/mol) of Kr/Xe@GN obtained by using B3LYP+D3/def2-SVP. Structures correspond to Figure 4g-m.

| **structure** | **Kr** | **Xe** |
| --- | --- | --- |
| **g** | -3.81 | -6.78 |
| **h** | -4.31 | -7.82 |
| **i** | -4.56 | -9.50 |
| **j** | -7.32 | -10.13 |
| **k** | -7.41 | -10.25 |
| **l** | -9.75 | -13.81 |
| **m** | -9.83 | -15.10 |
